# Identification and Validation of New Alleles of *FALSIFLORA* and *COMPOUND INFLORESCENCE* Genes Controlling the Number of Branches in Tomato Inflorescence

**DOI:** 10.3390/ijms18071572

**Published:** 2017-07-20

**Authors:** Huan Zheng, Saneyuki Kawabata

**Affiliations:** Institute for Sustainable Agro-Ecosystem Services, Graduate School of Agricultural and Life Sciences, The University of Tokyo, Midori-cho, Nishitokyo, Tokyo 188-0002, Japan; huanzheng1988@hotmail.com

**Keywords:** tomato, QTL-seq, branch number, *FALSIFLORA*, *COMPOUND INFLORESCENCE*

## Abstract

The architecture of inflorescences shows extensive diversity in both branching frequency and flower number, which eventually, determines agricultural productivity. In this study, F_2_ (second filial) populations derived from a cross between *Solanum lycopersicum* 10AS111A (highly-branched inflorescence) and the *S. pimpinellifolium* PI124039 (inflorescence having a single branch) were used to decipher the genetic control of branch number (BN) of inflorescence in plants bearing small-sized tomato fruits. The segregation ratio of single- and moderately-branched types to the highly-branched type was significantly different from 3:1 but not different from 15:1 at *p* < 0.05, suggesting that more than one gene controls the branch number of the inflorescences. Through genome-wide comparison of single-nucleotide polymorphism (SNP) profiles between the highly-branched type bulk and the single-branch type bulk constructed using the F_2_ plants, we identified a major quantitative trait locus (QTL) on chromosome 3 (58.75–61.4 Mb) and a minor QTL on chromosome 2 (32.95–37.1 Mb), which explained 15.7% and 6.1% of the BN variation, respectively. *FALSIFLORA (FA)* and *COMPOUND INFLORESCENCE (S)* genes, located in the QTL peak regions, caught our attention. Sequence comparison of the *FA* and *S* genes and their promoter regions from the two parental lines revealed that both contain missense mutations in the coding regions. Segregation analysis of *FA* and *S* alleles by high-resolution melting (HRM) method confirmed that alleles for both genes from 10AS111A significantly increased the BN and the size of inflorescence. In conclusion, we propose that SNPs in coding sequences might cause changes in the function of *FA* and *S* genes, which might be important determinants of BN.

## 1. Introduction

Tomato (*Solanum lycopersicum* L.) is an agriculturally and economically important vegetable crop worldwide. The architecture of tomato inflorescence varies greatly from a simple type having a single inflorescence branch to highly branched type having dozens of inflorescence branches. The branch number (BN) of the inflorescence is an important trait that determines the final number of fruits produced per inflorescence, which then influences the yield. In small-fruited tomato production, moderately-branched inflorescence structure has an advantage for producing a sufficient number of fruits and preventing possible yield reduction due to the small fruit size [1]. When the inflorescences are extremely branched, an intense fruit competition within inflorescence may be induced and this causes insufficient fruit development and, thus, a reduction in fruit quality.

On the basis of the inflorescence meristem (IM) growth habits, two major architectural types of inflorescences can be distinguished in tomato: monopodial and sympodial [2]. In monopodial development, growth of the inflorescence axis is maintained by the indeterminate development of IMs, which continuously initiate lateral (axillary) branches or flowers. On the other hand, in sympodial development, growth of the IM is terminated multiple times as IM transitions to floral meristem (FM), and regeneration of the axis occurs through laterally initiated IM [2,3]. According to these developmental habits, the process of forming variable inflorescence architecture was considered to be affected by the timing of the transition of IM into FM [3]. If only one lateral IM is produced before the apical IM transitions to FM, the inflorescence will not branch and form a single branch structure. If multiple IMs are produced before the transition, the inflorescence axil will be bifurcated. If no IM produced before the transition, the inflorescence elongation will be terminated.

Previous studies showed that the highly-branched phenotype of a tomato variety ‘Wonder of Italy’ resulted from a mutation in *COMPOUND INFLORESCENCE* (*S*, homolog of *WUSCHEL HOMEOBOX 9*, *WOX9*). The segregation of simple-branched type to highly-branched type in F_2_ (second filial) generations derived from a cross between ′Wonder of Italy′ and a simple type cultivar ′Lister’s Prolific′ was close to a 3:1 ratio, suggesting that the highly-branched inflorescence is controlled by a recessive allele [4,5]. The *S* gene allele, *s-classic*, found from ‘Wonder of Italy’ was also identified in 22 domesticated highly-branched varieties [5].

Mutations in other two genes, *ANANTHA* (the homolog of *UFO*) and *FALSIFLORA* (*FA*, homolog of *LEAFY*, *LFY*) also caused frequent inflorescence branching, but fertile flowers were replaced by cauline leaves or bracts, and no visible floral structures were formed in the *an* and *fa* mutants. Further studies in these three genes suggested that *S* was required for the maintenance of IM activity, while *AN* and *FA* formed a complex to specify flower formation [5]. Therefore, mutations in these genes will delay (*s*) or block (*an* and *fa*) the IM transition to FM, leading to branched inflorescences.

Although a precise control of inflorescence branching is required for optimizing the fruit production in small-fruited tomatoes, their molecular mechanisms remain obscure. The parental line 10AS111A was derived from a ′double truss′ cultivar ′Sicilian Rogue′, which was kindly provided by the Musashi Plant Breeding Corp. We found that although 10AS111A showed a highly-branched inflorescence phenotype, it did not carry the *s-classic* allele by sequencing analysis. This observation suggested that there should be another *S* allele or another mechanism than *s-classic*, that causes inflorescence branching.

In this study, we used the restriction site associated DNA (RAD) sequencing-based quantitative trait loci sequencing (QTL-seq) [6] approach in the bulks of F_2_ generation to identify the major genomic regions harboring QTLs associated with BN in tomato progeny of 10AS111A. The integration of QTL-seq with allele-specific association analysis delineated two candidate genes and new allelic variants within the QTL intervals governing BN of tomato inflorescence.

## 2. Results

### 2.1. Variation and Inheritance of BN in the Two Parental Lines, F_1_, and F_2_ Populations

**Figure 1:**
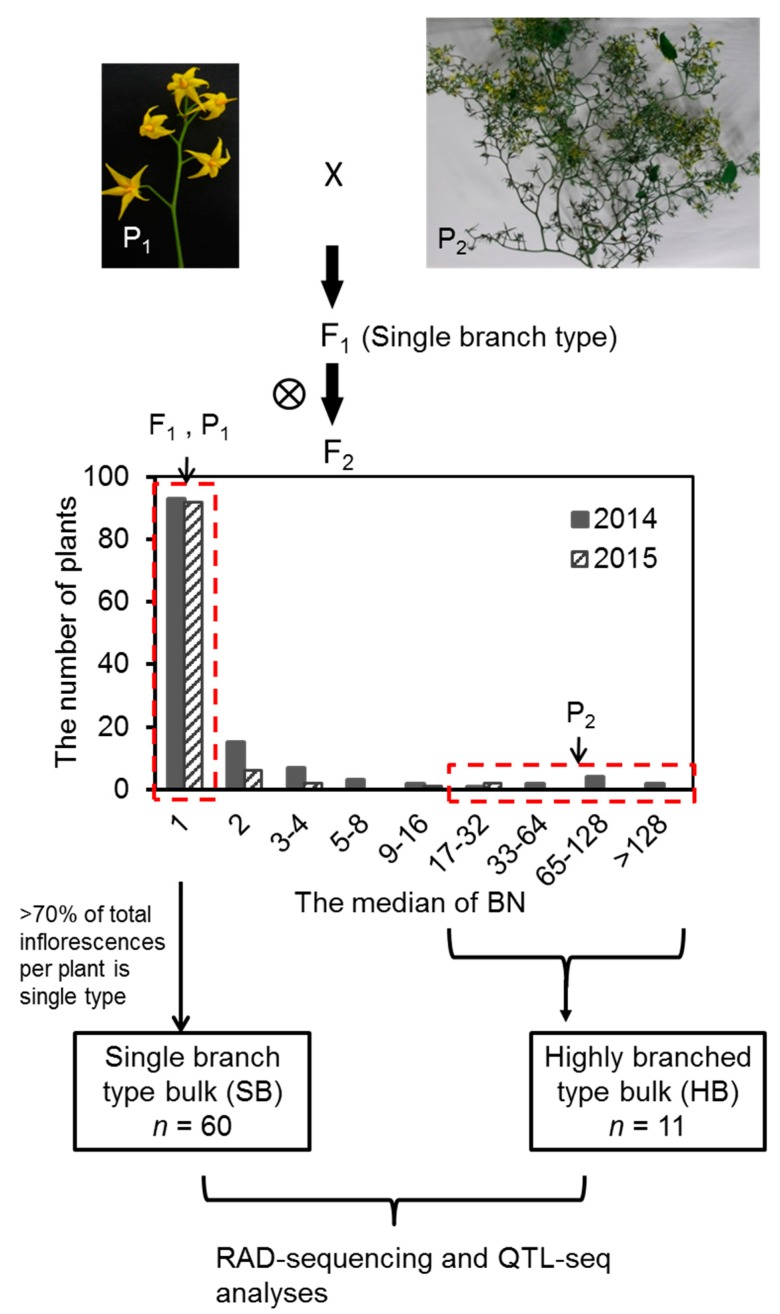
Flowchart of the BN QTL detection. The F_2_ populations were constructed from a cross between highly-branched inflorescence type 10AS111A and the single-branch inflorescence type PI124039. Because the BN of inflorescence was not consistent along the stem within one plant, we defined the single branch type as the plant in which the median of BN was one and more than 70% of the inflorescences were single-branch inflorescences. If the median of BN was more than 16, they were categorized as highly-branched plants. Sixty plants with single -ranch inflorescence and 11 plants with highly-branched inflorescence grown in 2014 were pooled and used for RAD sequencing and QTL-seq analysis. A histogram of F_2_ populations across the years shows the number of plants bearing different numbers of branched inflorescences is shown. P_1_: *Solanum pimpinellifolium* PI124039; P_2_: *S. lycopersicum* 10AS111A; F_1_: The F_1_ derived from P_1_ and P_2_. The arrows indicate the BN of F_1_, P_1_ and P_2_, averaged for the two-year experiments. ⊗ means self-crossing.

**Figure 2:**
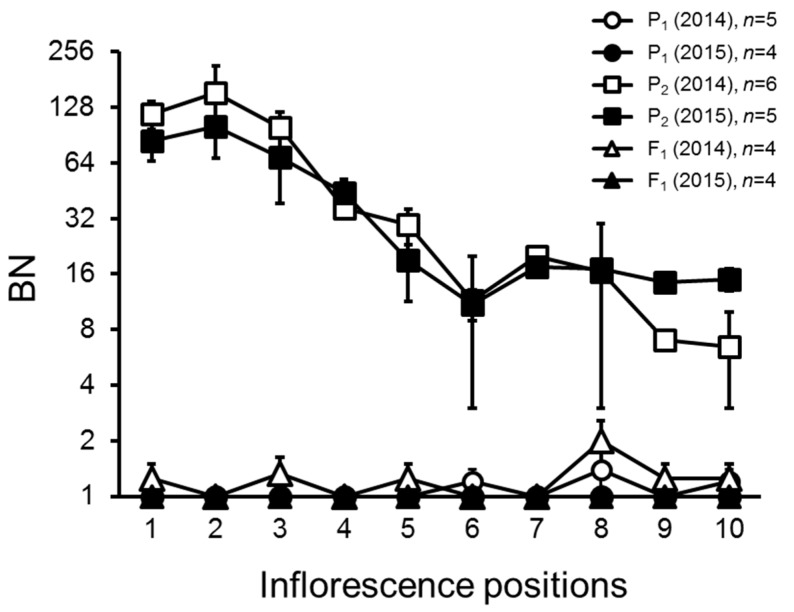
Effects of the inflorescence position and different growing seasons on the variation of BN in the F_1_, PI124039, and 10AS111A lines. Values are given as mean ± SD. P_1_: *Solanum pimpinellifolium* PI124039; P_2_: *S. lycopersicum* 10AS111A; F_1_: the F_1_ is derived from P_1_ and P_2_.

Inflorescences of 10AS111A were highly-branched, especially at the base node position on the plant, while the inflorescence of PI124039 and the F_1_ plants were of the simple-type, having one to a few inflorescence branches (Figure 1 and Figure 2).

Frequency distribution of BN among test materials in two years is presented in Figure 1. In both 2014 and 2015 experiments, most of the F_2_ individuals had single branch or only slightly branched inflorescences. The number of highly-branched type plants (>16 branches) was 11 and two in 2014 and 2015, respectively (Figure 1). The observed segregation ratio of single branch or moderately-branched type to highly-branched type was significantly different from 3:1 but not different from 15:1 at *p* < 0.05 (Table S1). The broad-sense heritability (H^2^) of the BN in two-year experiments was 62.8% and 74%, respectively (Table S2). The trait showed both high values of skewness and kurtosis, suggesting the major single branch allele in PI124039 was dominant (Table S2 and Figure 1).

### 2.2. Joint QTL-seq and Bulk Segregant Analysis Used to Identify QTL Loci Controlling BN

**Figure 3:**
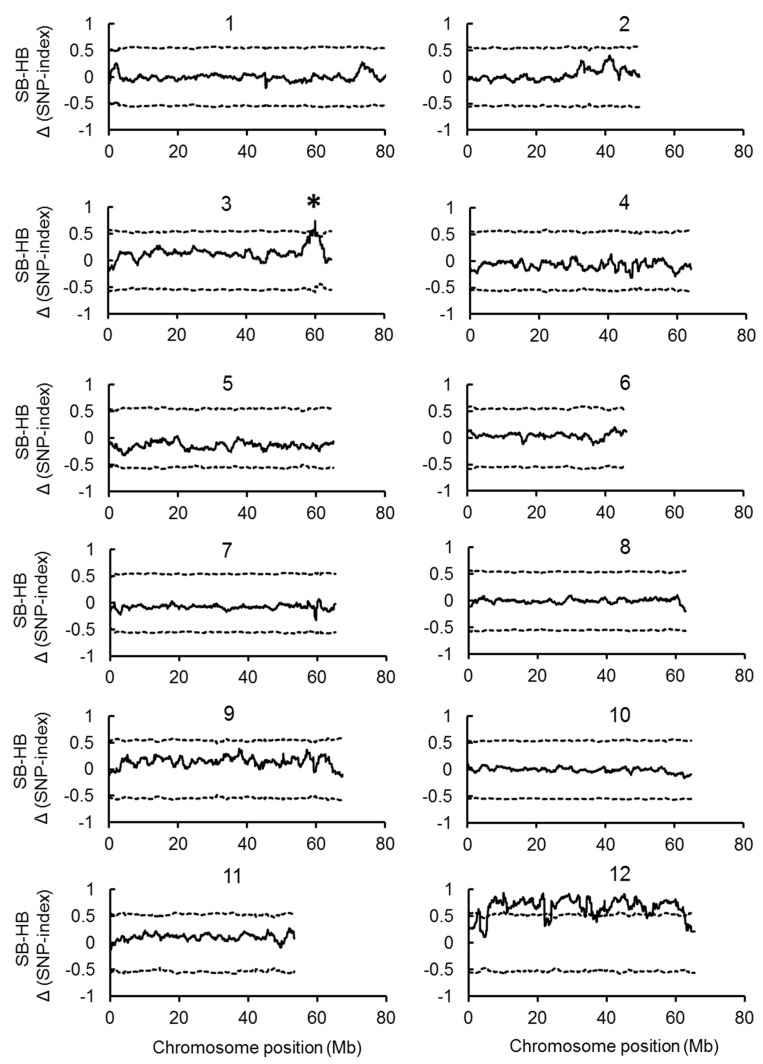
Identification of BN QTLs in tomato chromosome. The Δ (SNP-index) was calculated as SB’s SNP-index subtracted from HB’s SNP-index. The X-axis denotes the physical positions (Mb) on the tomato chromosomes. The Y-axis represents the Δ SNP-index, which has been estimated for 2 Mb physical intervals with a 50 kb sliding window. The dotted lines are the threshold value, which are plotted using the statistical confidence intervals under the null hypothesis of no QTL (*p* < 0.05) according to Takagi et al. [6]. One major genomic region (58.75–61.4 Mb, marked by an asterisk) harboring a robust BN QTL (BN3.1) was defined using the following criteria: SNP-index near 1 and 0 in SB and HB, respectively, and the confidence value of Δ above 0.5 (at a significance level *p* < 0.05).

Illumina high-throughput RAD sequencing of 10AS111A, SB, and HB generated 22–25 million 90 bp short sequence reads. Most of the bases (>97.8%) were of a high quality, with quality scores of at least 20 and a guanine and cytosine (GC) percentage around 36%. All the sequence date (two parental lines and two bulks) were submitted to the Sequence Read Archive (SRA) at NCBI (SRA accessions SRR5714430, SRR5714431, SRR5714432, SRR5714433) for unrestricted public access. These short reads from 10AS111A were mapped to the publically-available reference genome of tomato (*Solanum lycopersicum* SL2.40), then aligned 10AS111A genomic sequence was used as the reference sequence for detecting SNPs. A total of 15,640 SNPs were identified between SB and the reference genome. All these SNPs were mapped on the 10AS111A genome with an average depth of 12× (Figure S1). The SNP-index was calculated for each identified SNP. Average SNP-indices of SB and HB bulks were calculated for a 50 kb intervals by a 2 Mb sliding window, were plotted against the position of each sliding window on the 10AS111A genome (Figure S2). The Δ (SNP-index) was calculated as the differences between SNP-indices for SB and HB bunk, and plotted against the genome positions along with the SNP-indices of SB and HB (Figure 3).

A major QTL on chromosome 3 (58.75–61.40 Mb) was detected. In this region, the average SNP index of SB was higher than 0.8, but lower than 0.2 for HB. We obtained a significant value of Δ (SNP-index) for this peak, which was higher than 0.5 (*p* < 0.05) (Figure 3 and Table S3). In addition, there were two small peaks on chromosome 2 (32.95–37.10 Mb and 39.80–42.25 Mb). The Δ (SNP-index) value of the second peak reached 0.40. Although these regions were not significant at *p* < 0.05, we considered these regions as the candidates as well (Figure 3 and Table S3). We also used PI124039 as the reference sequence for detecting QTLs, although the peak of Δ (SNP-index) values were lower than those calculated using 10AS111A as the reference genome, and the peak region did not change (Table S3).

### 2.3. Identification of Candidate Genes for the BN Loci

**Figure 4:**
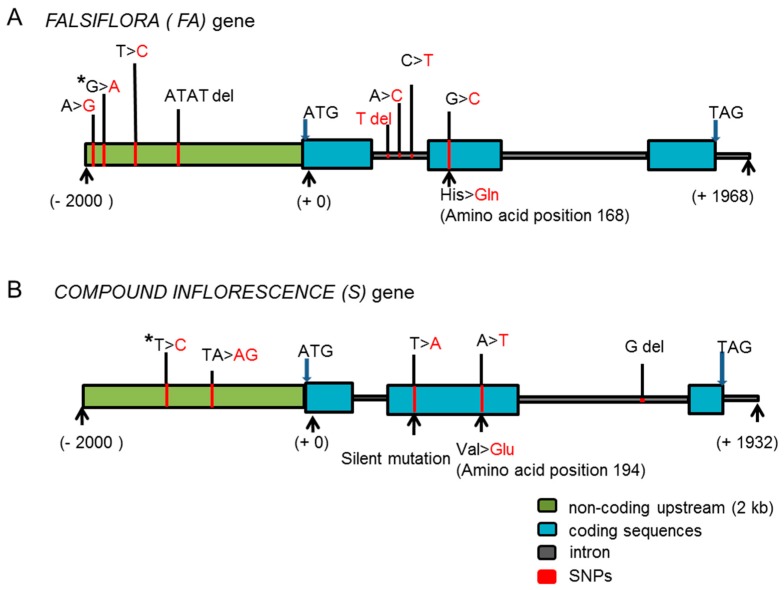
The structure and mutations of *FA* and *S* genes compared between the parents. (**A**) One amino acid substitution SNP (C/G, which changed the His in PI124039 to Gln in 10AS111A) identified in the second exon of the *FA* gene is indicated using an arrow; (**B**) two SNPs are located in the coding region, one a silent mutation, and the other changing Val in PI124039 to Glu in 10AS111A in the *S* gene. Exons are shown by blue boxes, introns are shown by grey lines, non-coding upstream regions (2 kb) are shown by green boxes, and the SNPs are shown by red lines. The SNPs in PI124039 are shown in black font and the one in 10AS111A is highlighted using red font. The SNPs marked by asterisk were designed as markers for HRM analysis of individuals of F_2_ populations. del: deletion.

Following the results of QTL-seq (Figure 3), we initially annotated hundreds of genes near the peak region on chromosome 3, using the National Center for Biotechnology Information (NCBI) database (http://www.ncbi.nlm.nih.gov/). The *FA* gene caught our attention due to prior knowledge of its function [7]. We re-sequenced the *FA* gene and its promoter sequence from PI124039 and 10AS111A. Seven SNPs and a small indel were revealed between the two parents. One of the SNPs in the middle of exon 2 (position chr_03:61167529 C/G) [8] changed the amino acid from histidine in PI124039 to glutamine in 10AS111A (Figure 4A).

We also analyzed the other two peak regions on chromosome 2 (32.95–37.10 Mb and 39.80–42.25 Mb), which were also predicted to harbor hundreds of genes. These regions included two genes which were reported to be involved in control of the IM identity, i.e., *S* and *AN* [5]. We re-sequenced these genes and the non-coding upstream sequences from P_1_ and P_2_, and identified four SNPs and a small nucleotide transition in the *S* gene (Figure 4B), but no polymorphism in the *AN* gene. Two SNPs in the *S* gene were located in the coding region, one was a silent mutation, and the other changed valine in PI124039 to glutamic acid in 10AS111A (position chr_02:36916232 A/T) [8] (Figure 4B).

### 2.4. Inflorescence Trait Variations Associated with FA and S Loci

**Figure 5:**
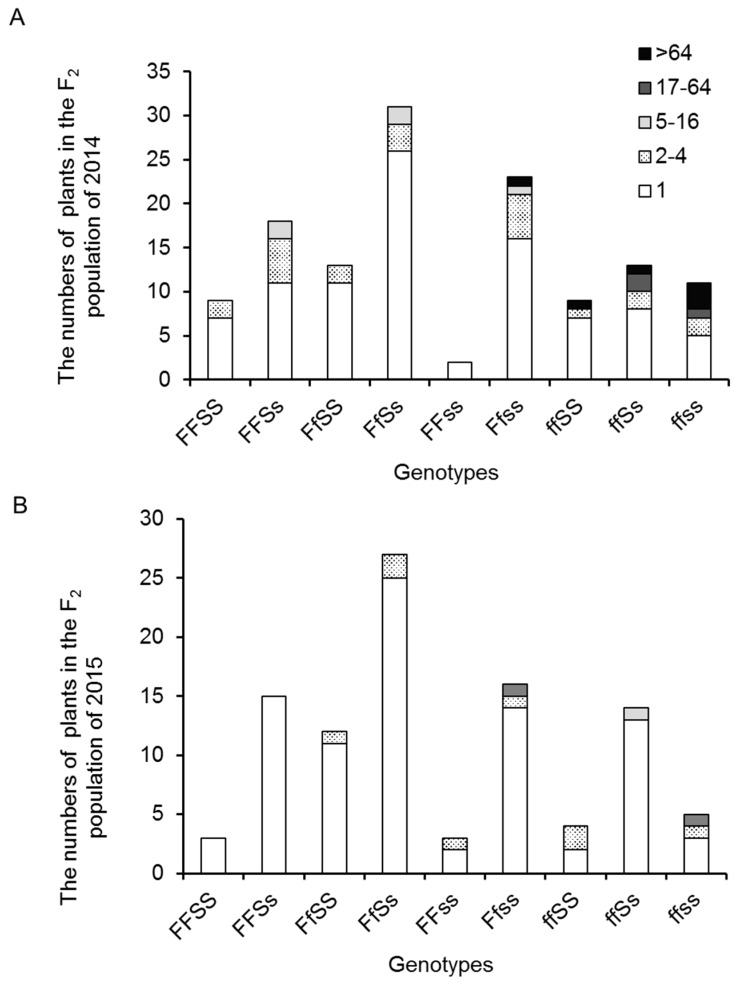
The relationship between BN and genotypes with reference to the candidate genes in F_2_ populations of 2014 (**A**) and 2015 (**B**). Plant branch type (defined by the median of BN of the plant) were categorized into 1, 2–4, 5–16, 17–64, and more than 64 branches. Frequency distribution of BN groups of the nine genotypes for the *FA* and *S* genes in the F_2_ population of 2014 is shown. *S/s* represents *S* gene allele; *F/f* represents *FA* gene allele. *S* and *F* alleles are derived from PI124039; *s* and *f* alleles are derived from 10AS111A.

**Figure 6:**
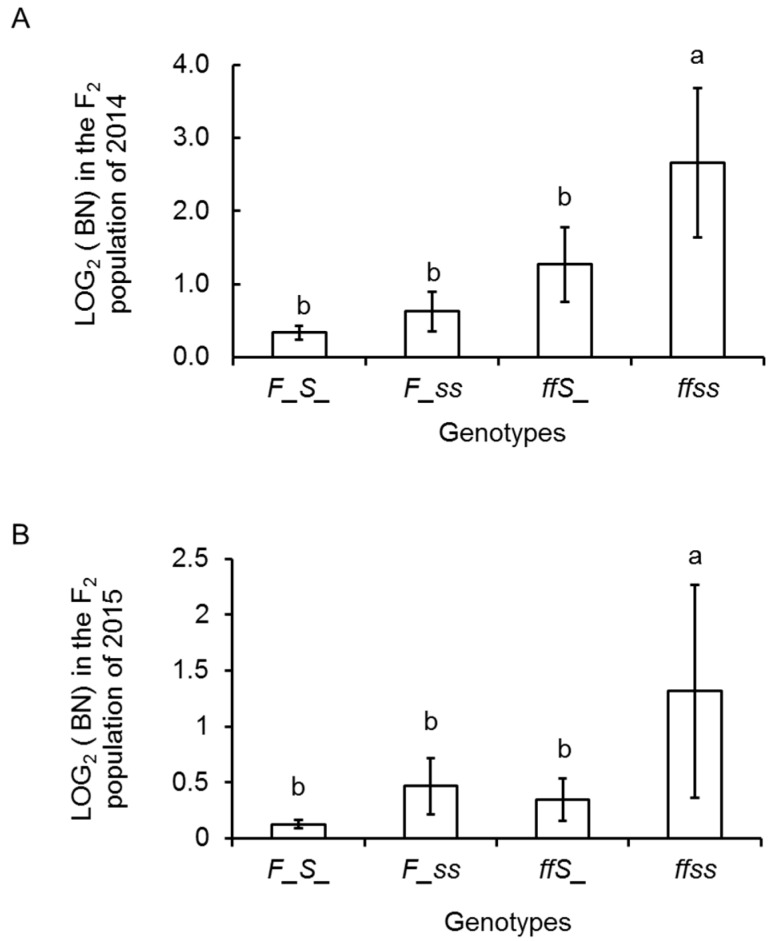
Differences in LOG_2_(BN) values among the four genotypes were compared using Tukey’s HSD test; values are given as mean ± SD. (**A**) LOG_2_(BN) in the F_2_ population of 2014; *n* = 71 (*F_S*_), *n* = 25 (*F_ss*), *n* = 22 (*ffS*_), *n* = 11 (*ffss*); and (**B**) LOG_2_(BN) in the F_2_ population of 2015. *n* = 57 (*F_S*_), *n* = 19 (*F_ss*), *n* = 18 (*ffS*_), *n* = 5 (*ffss*). The different letters indicate significance at *p* < 0.05. *S/s* represents *S* allele; *F/f* represents *FA* allele.

Two SNP markers from the *FA* and *S* genes were used to genotype the F_2_ populations. In 129 F_2_ individuals of 2014, where the HRM analysis was conducted (Figure S3), the segregation ratio of the homozygote for the 10AS111A allele: heterozygote for F_1_ alleles: homozygote for the PI124039 alleles was 30:69:31 (Chi square = 0.46, non-significant at *p* < 0.05) for the *FA* locus and 33:61:36 (Chi square = 0.25, non-significant at *p* < 0.05) for the *S* locus. In 99 F_2_ individuals of 2015, the segregation ratios were 21:56:22 (Chi square = 1.73, non-significant at *p* < 0.05) for the *FA* locus and 20:56:23 (Chi square = 1.64, non-significant at *p* < 0.05) for the *S* locus. Thus, both data fit well with the expected ratio (1:2:1) for single locus Mendelian segregation. Combining the data obtained from *FA* and *S* markers, the F_2_ populations were classified into nine genotypes. Figure 5 shows the BN distribution of various genotypes. All the highly-branched individuals were homozygous for the *FA* and/or *S* allele from 10AS111A. As expected, the genotype *ffss* showed the highest value of LOG_2_(BN) and genotype *F_S_* presented the lowest value for both F_2_ populations (Figure 6).

The non-parametric test for *S* and *FA* genotypes in the BN groups also established significant association between marker and phenotype (Figure S4), with the *FA* locus explaining 15.7% and *S* locus explaining 6.1% of the BN variation in the F_2_ population (Figure S4A) of 2014.

Associations between other inflorescence traits and *FA* and *S* genotypes were assessed by ANOVA and non-parametric tests. Since BN determined FN and DW to some extent, both of them were significantly associated with *FA* and *S* loci (Figure S4B,C). Flowering time was significantly associated with the *S* locus, which showed correlation with the developmental stage (NLF), but was not significantly associated with the actual time taken (DA) (Figure S4D,E). We also analyzed floral traits by measuring the sepal and petal length. Only the *FA* locus was found significantly associated with SL (Figure S4F,G). MBD and MBL were also significantly associated with the *S* locus; however, no significant association was found with the *FA* locus (Figure S4H,I).

## 3. Discussion

### 3.1. The New Allele Identified for the FALSIFLORA Gene Is the Candidate Gene Involved in the Control of BN in Tomato

In this study, we constructed the F_2_ population derived from a cross between a highly-branched inflorescence type and single-branch type tomatoes. The observed segregation ratio of single-branch or moderately-branched type to highly-branched type was significantly different from 3:1 but not different from 15:1 at *p* < 0.05 (Table S1), suggesting that more than one gene regulated BN. By the RAD sequencing based QTL-seq analysis, we identified and mapped one major genomic region harboring a BN QTL on chromosome 3 (Figure 3). We found the *FA* gene located in the genomic region adjacent to the QTL.

Molinero-Rosales et al. [7] were the first to identify the *FA* mutant of tomato. Flowers of the *fa* mutant were altered into secondary inflorescence axis, producing highly-branched inflorescences with cauline leaves or bracts. Sequence analysis of FA showed that the predicted protein shared 80% and 70% identity with FLO and LFY proteins, respectively. The *LFY* gene of *Arabidopsis* and its homologs encode a master transcription factor that plays pleiotropic roles in regulating the transition from the vegetative to the reproductive phase, the floral fate of meristems, the flowering time, and the arrangement of flowers [1,9,10,11,12]. Due to the important role of LFY in the reproductive transition, even weak mutant alleles caused a complete transformation of at least a few flowers into shoots [13]. Most *FLO/LFY* single mutants in dicots showed an increase in branching due to the conversion of flowers into shoots, suggesting that the *FLO/LFY* mutant suppressed branching by promoting flower development [10,13,14]. Additionally, LFY activity was partly dependent on environmental and internal factors, such as light conditions and plant age, and could be induced by exogenous GA_3_ treatment [15,16,17,18].

Sequence comparison between *FA* alleles from the two parents revealed a clear SNP in the coding region, which changed the histidine (positively-charged) in PI124039 to glutamine (uncharged) in 10AS111A (Figure 4A). Changes in the polarity of amino acids may affect protein function [19]. In addition, the HRM study was designed to classify genotypes of the 129 F_2_ individuals of 2014 population. The *FA* genotypes were found to be significantly (*p* < 0.05) associated with BN, FN, DW, and SL (Figure S4). Therefore, we propose that the new allele of *FA* is the candidate gene for regulating the inflorescence architecture, primarily through control of the BN in plants bearing small sized tomatoes.

### 3.2. Effects of FA and S Depend on the Genetic and Environmental Background

Molinero-rosales et al. [7] compared the *FA* cDNA sequences in mutant plants with the wild-type and observed two differences. One was a 3 bp insertion (TAG) at position 479, which gives rise to a protein with an additional valine residue at position 158. Another change was a deletion of 16 bp in exon 2 resulting in a frameshift mutation. The deletion leads to the mutant plant becoming sterile, as it never produced flowers, even during the late developmental stages [7] (Figure S5). In this study, only one residue was changed in the predicted FA protein from 10AS111A compared with the wild-type PI124039 (Figure 4A and Figure S5). Both 10AS111A and the F_2_ lines, which were homozygous for the *FA* allele from 10AS111A, were highly branched, lacking the determinate growth characteristic of tomato inflorescence, appearing very similar to the *FA* mutant plant except for the production of mature flowers. All the F_2_ lines and 10AS111A were fertile and produced normal flowers. This may be because the mutation of *FA* in 10AS111A is only slightly deleterious so that it just delayed the IM maturation but did not inhibit the IM transition to FM.

Lippman et al. [5] identified three independently mutated alleles of *S* (*s-classic*, G > D; *s-multiflora*, I > F, and Rose Quartz Multiflora) (Figure S6), which might be responsible for a major portion of the diversity in tomato inflorescence architecture. In this study, the SNP in the second exon of *S* (outside the homeodomain region) changed a valine in PI124039 to a glutamine in 10AS111A at position 194 (Figure 4B and Figure S6). Since this new allele of *S* from 10AS111A was significantly correlated with BN, FN, NLF, MBL, MBD, and DW in the F_2_ generation (Figure S4), the allele may increase the size of inflorescence. When compared with the *s-classic* and *s-multiflora* alleles, in which an amino acid in the homeodomain was changed [5], the new *S* allele identified in this study had smaller effect, and the effect was smaller than the *FA*. This explains the insignificant QTL-seq results obtained for chromosome 2 (Figure 3).

An interesting observation was the higher expression level of *S* gene in *FA* mutant inflorescences of wild-type plants and the enhancement in *FA* expression level in *S* mutant compared with the wild-type plant [5]. In the present study, we observed that all the highly-branched plants are homozygous for the *FA* or *S* alleles from 10AS111A. Plants homozygous for alleles of both the genes from 10AS111A have the highest ratio of highly-branched phenotype (Figure 5). Altogether, these results indicate that *FA* and *S* may play a common role during inflorescence development or may cooperatively play important roles in the development of inflorescence architecture.

## 4. Materials and Methods

### 4.1. Phenotyping of Mapping Population for Inflorescence Traits

The F_2_ population was constructed from a cross between the *Solanum lycopersicum* accession 10AS111A (highly-branched inflorescence; P_1_; kindly provided by the Musashi Plant Breeding Corp., Saitama, Japan) and the *S. pimpinellifolium* accession PI124039 (inflorescence having a single branch; P_2_). The sizes of F_2_ population used in 2014 and 2015 were 129 and 103, respectively. Seeds were sown in 2 L plastic pots filled with 3:1 mixture of granular clay soil and peat-based soil mix (Metro-mix 360, Hyponex, Osaka, Japan). In 2014, the F_2_ plants were sown on 30 May and grown in a greenhouse (University of Tokyo, Tokyo, Japan) from seedling stage to the end of the experiment (10 November). In 2015, the F_2_ plants were sown on 19 January and grown in a room controlled at 28/23 °C (day/night) with natural light for one month and then transferred to the same greenhouse until 30 August. The plants were trimmed as single stem by regularly removing lateral shoots, and fertilized weekly with liquid fertilizer Hyponex (Hyponex Japan Corp., Ltd., Osaka, Japan).

The first to tenth inflorescences of each plant were harvested on maturation of the first fruit of that inflorescence. A ‘branch’ of inflorescence was defined as a stretch of inflorescence axis arising from a parent branch, having no daughter branches, and bearing more than two flowers. After the inflorescences were collected, the number of leaves preceding the first inflorescence (NLF), the days from sowing to the first flower anthesis (DA), the BN per inflorescence, flower number per inflorescence (FN), the length of the main inflorescence branch (MBL), the diameter of the main inflorescence branch at the basal position (MBD), sepal length (SL), and petal length (PL) were recorded. After taking these measurements, the inflorescence axis was dried at 80 °C for 24 h and the dry weight (DW) was determined. Owing to the skewed distribution of BN, it was log transformed before analysis. The broad-sense heritability (H^2^), the percentage of phenotypic variance explained (PVE) by each QTL, and the frequency distribution and association of quantitative phenotypes with *FA* and *S* alleles were analyzed following the methods described by Das et al. [20] and Bomblies et al. [21].

We performed a post hoc pairwise test using Tukey’s honestly significant difference (HSD) test when Levene’s statistic was not significant but analysis of variance (ANOVA) indicated a significant group effect. Additionally, the non-parametric Jonckheere-Terpstra test was performed using SPSS 16.0.0 (SPSS Inc., Chicago, IL, USA).

### 4.2. Mapping and QTL Analyses

Genomic DNA was isolated from freshly harvested leaves (about 0.02 g) of the P_1_, P_2_, F_1_, and F_2_ plants from the 2014 experiment, using the Plant Genomic DNA Kit (TIANGEN Biotech, Beijing, China) following the manufacturer’s instructions. For QTL-seq [6], two DNA bulks were prepared. The single-branch (SB) bulk was prepared by mixing equal amounts of DNA from 60 single branches (the median of BN was 1 and >70% of the inflorescences were single branch type within the plant) and the highly-branched bulk (HB) was constructed by mixing equal amounts of DNA from 11 highly-branched (BN > 16) inflorescences of F_2_ plants from the 2014 experiment. DNA isolated from the two bulks and two parental lines were sent to BGI-Japan (Kobe, Japan) for RAD sequencing (Figure 1).

Adaptor sequences, low-quality reads, and duplicate reads were trimmed and filtered prior to analysis. The short reads obtained from the two DNA-bulks were aligned against the tomato genome sequence (*Solanum lycopersicum* SL2.40) using BWA software [22]. SAM/BAM files were converted after alignment and SNP-calling was performed by SAM tools software [23]. We defined one of the parents as the reference sequence for QTL-seq analysis. The depth of the SNPs which were used for mapping means how many reads, on average, are likely to be aligned at a given reference base position. It can be calculated from the length of the original genome (G), the number of reads (N), and the average read length (L) as N × L/G. An SNP-index is the percentage of short reads harboring the SNP that are different from the reference sequence. We excluded SNPs with SNP-index < 0.2 for all SNP positions, which maybe false positives due to genomic repeat sequence, sequencing or alignment errors. Δ (SNP-index) was obtained by subtraction of the SNP-index of the HB from that of the SB [6,24]. We only choose the SNPs that Δ (SNP-index) have been detected in both bulks of DNAs. SNP-index and Δ (SNP-index) were calculated to identify candidate regions for BN QTL. The SNP-index graph was generated by sliding window analysis with a 2 Mb window size and 50 kb increment. Significant peak of Δ (SNP-index) at *p* < 0.05 level were obtained by QTL-seq analysis [6].

### 4.3. Sequence Analysis of Candidate Genes and Single-Nucleotide Polymorphism (SNP) Genotyping of F_2_ Individuals

We investigated the genomic DNA sequences of the candidate genes from the two parental lines. Primers for amplifying the *FA* and *S* genes and their non-coding upstream regions (2 kbp) were designed using the ‘Pick Primers’ tool (http://www.ncbi.nlm.nih.gov) (Table S4). The DNA sequences of the amplicons (<700 bp in length) were determined by Sanger sequencing (performed at FASMAC, Midorigaoka, Japan). DNAMAN (Version 5.2.2.0, Lynnon Biosoft, San Ramon, CA, USA) [25], and SeqMan (DNASTAR, Madison, WI, USA) [26] software packages were used for contig assembly and alignment. Single-marker QTL analysis in the F_2_ generations of 2014 and 2015 were performed by high-resolution melting (HRM) method [27]. HRM analysis was performed using a CFX-Connect (Bio-Rad Laboratories, Inc., Hercules, CA, USA). PCR was carried out in a 10 μL volume containing 5 μL SsoFast Eva green supermix (Bio-Rad Laboratories, Inc.), 5 μM primer, and 5 ng of genomic DNA. PCR parameters before melting curve acquisition were: one cycle of initial denaturation at 95 °C for 2 min, 40 cycles of 95 °C for 5 s, and 60 °C for 30 s. The melt step of HRM analysis was performed with increment of 0.5 °C 15 s from 55 to 95 °C.

## Supplementary Materials

Supplementary materials can be found at www.mdpi.com/1422-0067/18/7/1572/s1.
