# Supplementary material for: Identification and Validation of New Alleles of FALSIFLORA and COMPOUND INFLORESCENCE Genes Controlling the Number of Branches in Tomato Inflorescence
Source: Int J Mol Sci. 2017 Jul 20;18(7):1572. doi: 10.3390/ijms18071572 (PMC5536060; doi:10.3390/ijms18071572)
Supplement: Supplementary file 1 [file ijms-18-01572-s001.zip › ijms-203349-final edition supplementary/ijms-203349-supplementary figures.pdf]

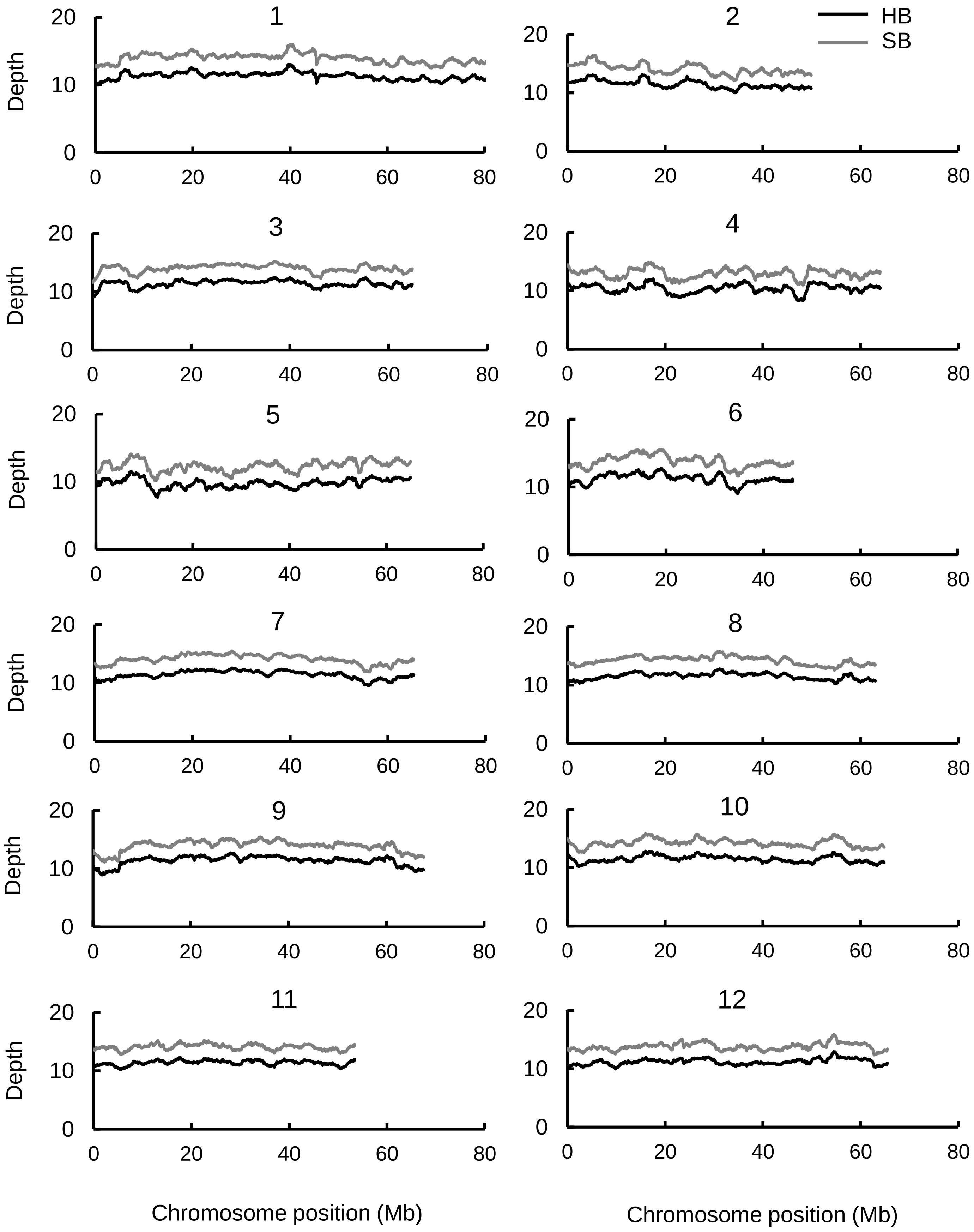

Figure S1. SNPs depth of whole genome. Dark line, depth of highly branched bulk (HB). Grey line, depth of single branch bulk (SB).

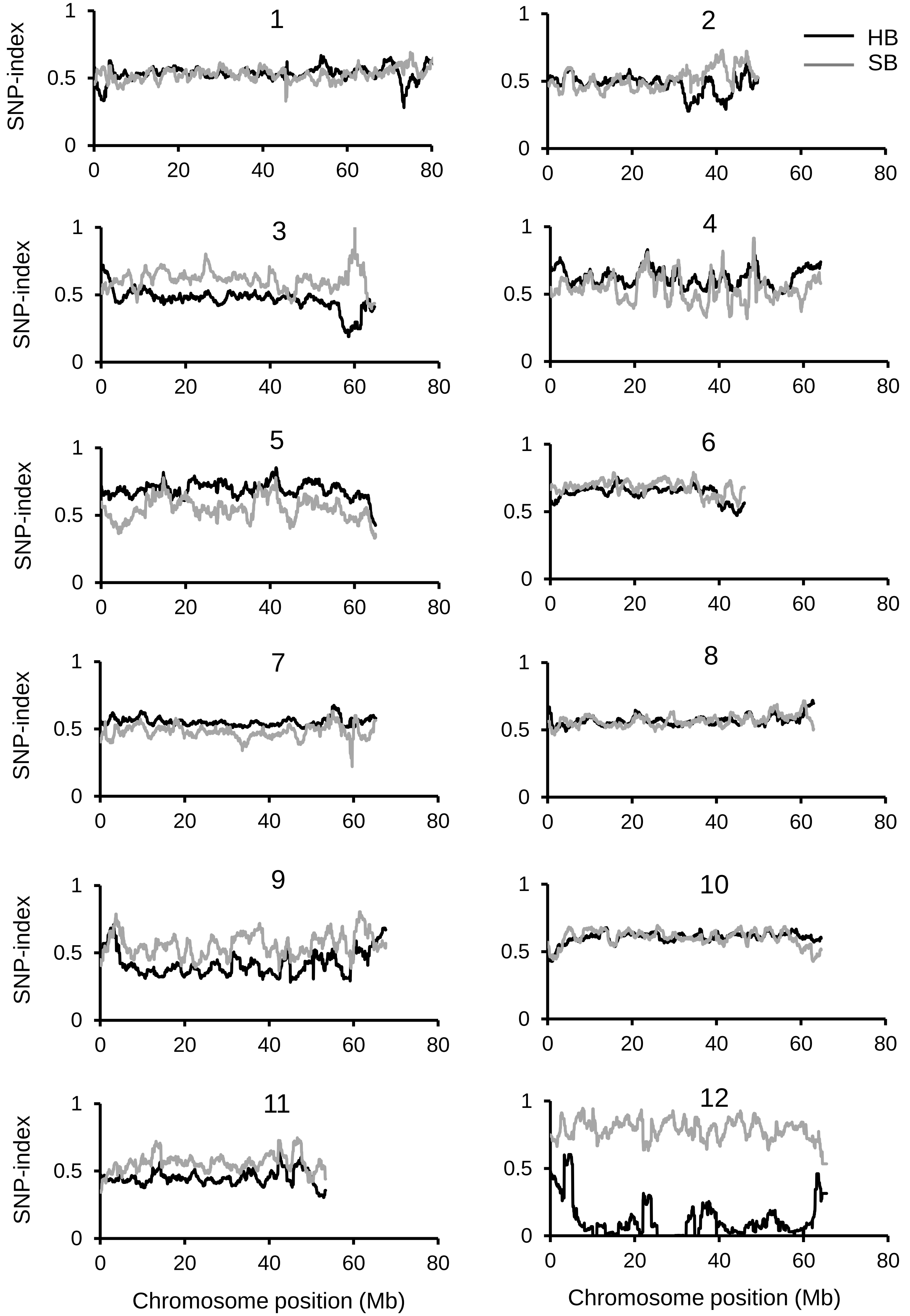

Figure S2. SNP-index graphs depict the single branch bulk (SB) and highly branched bulk (HB) generated from QTL-seq study. The X-axis denotes the physical positions (Mb) on tomato chromosomes. The Y-axis represents the SNP-index, which has been estimated for 2 Mb physical intervals with a 50 kb sliding window.

FA gene's marker

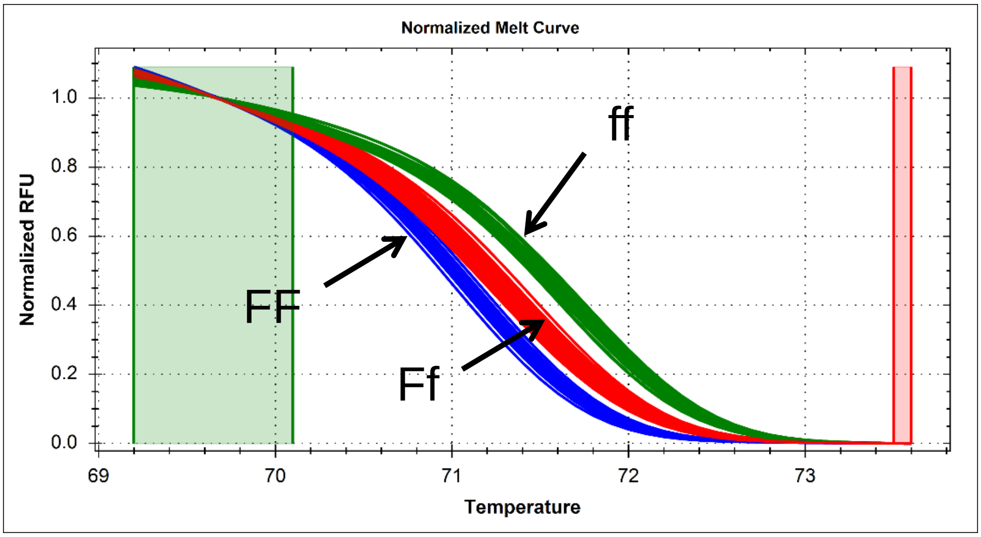

S gene's marker

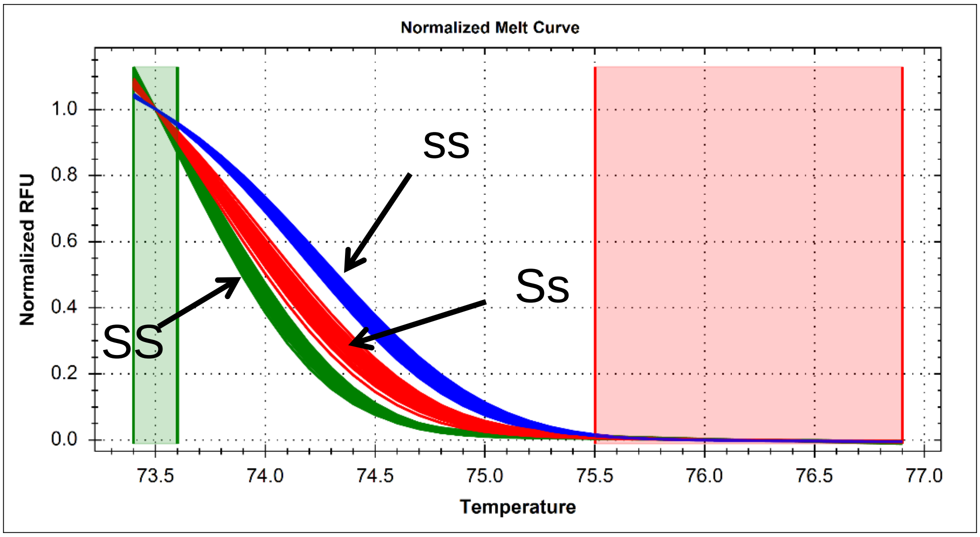

Figure S3. Genotyping of *FA* and *S* in F_2 individuals by HRM analysis. S/s represents *S* gene marker; F/f represents *FA* gene marker. Three genotypic classes of *S* and *FA* genes: SS/FF alleles, homozygous, from PI124039; ss/ff alleles, homozygous, from 10AS111A; Ss/Ff alleles, heterozygous, from F_1.

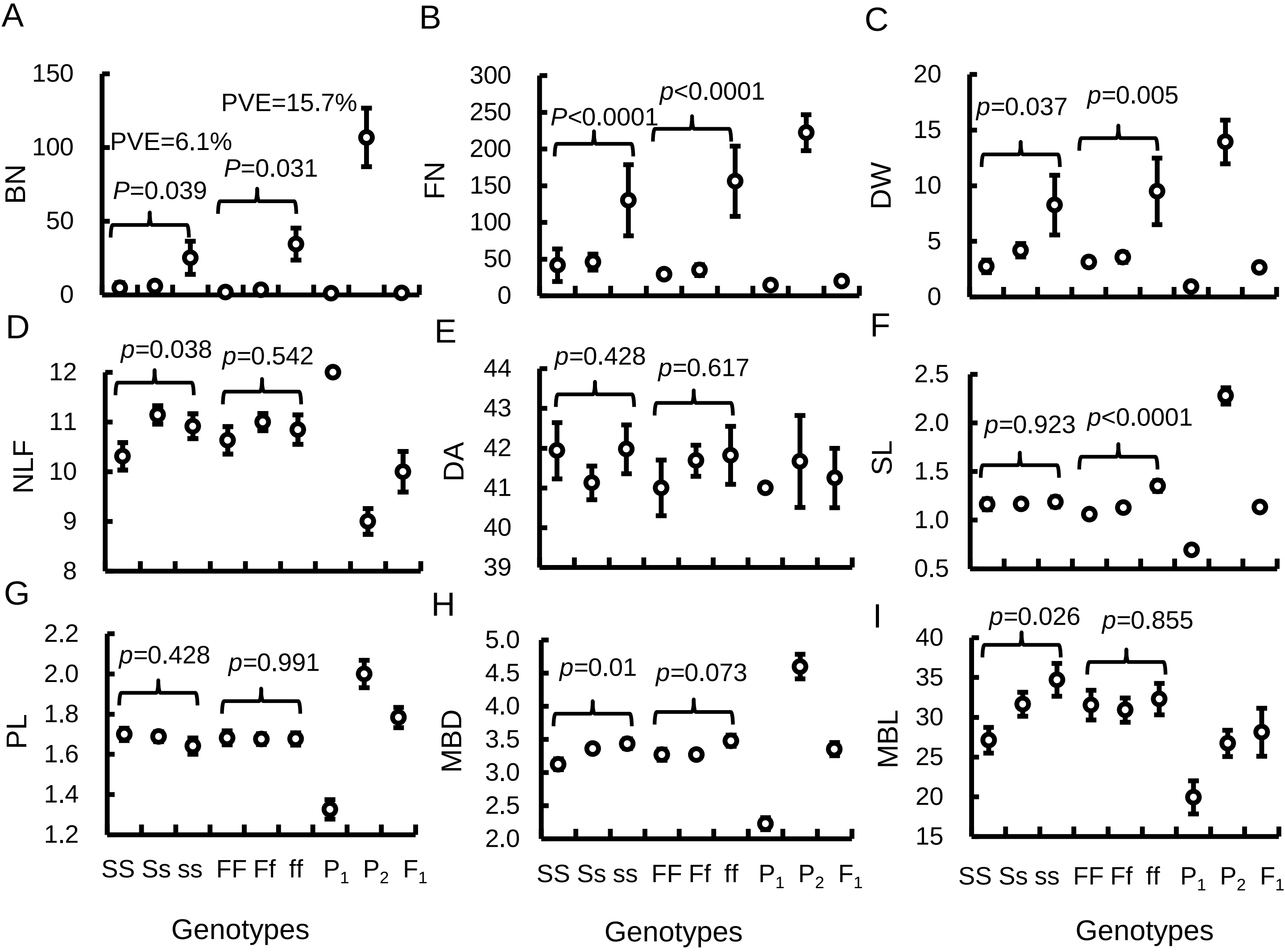

Figure S4. Quantitative effects associated with the *FA* and *S* genotypes. (A) The branch number per inflorescence (BN). (B) Flower number per inflorescence (FN). (C) Inflorescence dry weight (DW). (D) The number of leaves under the first inflorescence (NLF). (E) The days from sowing to the first flower anthesis (DA). (F) Floral trait, sepal length (SL). (G) Floral trait, petal length (PL). (H) The diameter of main inflorescence branch at the basal position (MBD). (I) The length of the main inflorescence branch (MBL). Trait values (mean ± SE) are plotted on the Y-axis of each graph, and genotypes with reference to *S* and *FA* in the parental lines and F_1 are plotted on the X-axis. The significance of variation in genotypes leading to variation in BN, FN, and DW was determined by Jonckheere-Terpstra test. Others traits and the *p* values for the associated ANOVA are also indicated. S/s represents *S* gene marker; F/f represents *FA* gene marker. Three genotypic classes of *S* and *FA* genes: SS/FF alleles, homozygous, from PI124039; ss/ff alleles, homozygous, from 10AS111A; Ss/Ff alleles, heterozygous, from F_1. P_1: *S. pimpinellifolium* PI124039; P_2: *S. lycoperscicum* 10AS111A; F_1: the F_1 derived from P_1 and P_2.

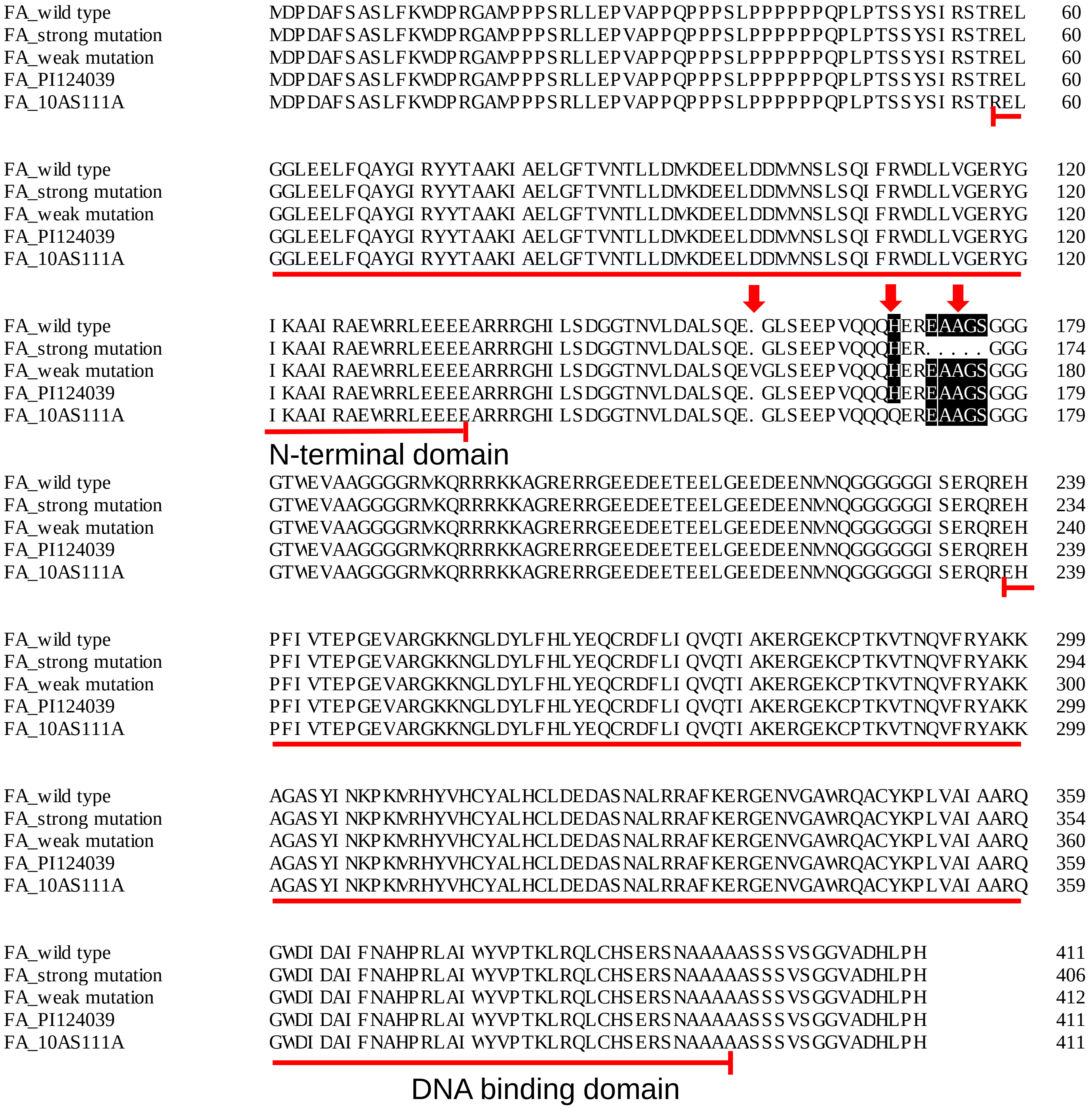

Figure S5. Comparison of the FA alleles. Different amino acids are shaded in black and indicated by red arrows. The domains are indicated below the alignment by red line. FA_wild type= *Solanum lycopersicum* Mill. FALSIFLORA (GenBank accession number AF197936).

```
S             MASSNRHWPSMFKSKPCNSHHHQWQHDINSSIIQQRPPCNPEERSPEPKPRWNPRPEQIR   60
S_CLASSIC     MASSNRHWPSMFKSKPCNSHHHQWQHDINSSIIQQRPPCNPEERSPEPKPRWNPRPEQIR   60
S_MULT        MASSNRHWPSMFKSKPCNSHHHQWQHDINSSIIQQRPPCNPEERSPEPKPRWNPRPEQIR   60
S_PI124039    MASSNRHWPSMFKSKPCNSHHHQWQHDINSSIIQQRPPCNPEERSPEPKPRWNPRPEQIR   60
S_10AS111A    MASSNRHWPSMFKSKPCNSHHHQWQHDINSSIIQQRPPCNPEERSPEPKPRWNPRPEQIR   60

S             ILEAIFNSGMVNPPRDEIRKIRAKLQEYGQVGDANVFYWFQNRKSRSKHKQRHLQAKAQQ  120
S_CLASSIC     ILEAIFNSDMVNPPRDEIRKIRAKLQEYGQVGDANVFYWFQNRKSRSKHKQRHLQAKAQQ  120
S_MULT        ILEAIFNSGMVNPPRDEIRKFRAKLQEYGQVGDANVFYWFQNRKSRSKHKQRHLQAKAQQ  120
S_PI124039    ILEAIFNSGMVNPPRDEIRKIRAKLQEYGQVGDANVFYWFQNRKSRSKHKQRHLQAKAQQ  120
S_10AS111A    ILEAIFNSGMVNPPRDEIRKIRAKLQEYGQVGDANVFYWFQNRKSRSKHKQRHLQAKAQQ  120

S             QHHNNNNNSSHQPIITSSSSSSDKSSPNSLTFSIGTSNVMDLLNSPTSSVNQQNYNEFLS  180
S_CLASSIC     QHHNNNNNSSHQPIITSSSSSSDKSSPNSLTFSIGTSNVMDLLNSPTSSVNQQNYNEFLS  180
S_MULT        QHHNNNNNSSHQPIITSSSSSSDKSSPNSLTFSIGTSNVMDLLNSPTSSVNQQNYNEFLS  180
S_PI124039    QHHNNNNNSSHQPIITSSSSSSDKSSPNSLTFSIGTSNVMDLLNSPTSSVNQQNYNEFLS  180
S_10AS111A    QHHNNNNNSSHQPIITSSSSSSDKSSPNSLTFSIGTSNVMDLLNSPTSSVNQQNYNEFLS  180

S             NEQPFFFTVQPPPVVPTHDHSAGFCFQDSSTFTPHSSSSGLLLNEWMGGISTQAPNNSKK  240
S_CLASSIC     NEQPFFFTVQPPPVVPTHDHSAGFCFQDSSTFTPHSSSSGLLLNEWMGGISTQAPNNSKK  240
S_MULT        NEQPFFFTVQPPPVVPTHDHSAGFCFQDSSTFTPHSSSSGLLLNEWMGGISTQAPNNSKK  240
S_PI124039    NEQPFFFTVQPPPVVPTHDHSAGFCFQDSSTFTPHSSSSGLLLNEWMGGISTQAPNNSKK  240
S_10AS111A    NEQPFFFTVQPPPVEPTHDHSAGFCFQDSSTFTPHSSSSGLLLNEWMGGISTQAPNNSKK  240

S             DENDKINLQSQLMSYTVTSTVSPLATTTIPTISHIQGVTVDPNDAGPTRSTVFINDVAFE  300
S_CLASSIC     DENDKINLQSQLMSYTVTSTVSPLATTTIPTISHIQGVTVDPNDAGPTRSTVFINDVAFE  300
S_MULT        DENDKINLQSQLMSYTVTSTVSPLATTTIPTISHIQGVTVDPNDAGPTRSTVFINDVAFE  300
S_PI124039    DENDKINLQSQLMSYTVTSTVSPLATTTIPTISHIQGVTVDPNDAGPTRSTVFINDVAFE  300
S_10AS111A    DENDKINLQSQLMSYTVTSTVSPLATTTIPTISHIQGVTVDPNDAGPTRSTVFINDVAFE  300

S             VGIGPFNVREVFGEDAVLIHSSGEPLITNEWGITIQPLQHGAFYYLLRTSSIASTHH     357
S_CLASSIC     VGIGPFNVREVFGEDAVLIHSSGEPLITNEWGITIQPLQHGAFYYLLRTSSIASTHH     357
S_MULT        VGIGPFNVREVFGEDAVLIHSSGEPLITNEWGITIQPLQHGAFYYLLRTSSIASTHH     357
S_PI124039    VGIGPFNVREVFGEDAVLIHSSGEPLITNEWGITIQPLQHGAFYYLLRTSSIASTHH     357
S_10AS111A    VGIGPFNVREVFGEDAVLIHSSGEPLITNEWGITIQPLQHGAFYYLLRTSSIASTHH     357
```

Homeodomain

C-terminal domain

Figure S6. Comparison of the S alleles. Different amino acids are shaded in black and indicated by red arrows. The domains are indicated below the alignment by red line. S= *Solanum lycopersicum* COMPOUND INFLORESCENCE (GenBank accession number NP_001234072).
