# Supplementary material for: Identification and Validation of New Alleles of FALSIFLORA and COMPOUND INFLORESCENCE Genes Controlling the Number of Branches in Tomato Inflorescence
Source: Int J Mol Sci. 2017 Jul 20;18(7):1572. doi: 10.3390/ijms18071572 (PMC5536060; doi:10.3390/ijms18071572)
Supplement: Supplementary file 1 [file ijms-18-01572-s001.zip › ijms-203349-final edition supplementary/ijms-203349-supplementary tables.docx]

**Supplementary Table 1.** Segregations of branch number in F_2_ populations.

|  | **Observed Segregation** | |  |  |
| --- | --- | --- | --- | --- |
| **Generation** | **Simple and Moderately Branched Type** | **Highly Branched type** | **χ^2^ (3:1)** | **χ^2^ (15:1)** |
| F_2_ (2014) | 120 | 9 | 21.63** | 0.12 |
| F_2_ (2015) | 101 | 2 | 27.48** | 3.26 |

** Significance at *p* < 0.01

**Supplementary Table 2.** Branch number (BN) of the parental lines, F_1,_ and the two F_2_ populations.

| **Year** | **10AS111A** | **PI124039** | **F_1_** | **F_2_** | | | | |
| --- | --- | --- | --- | --- | --- | --- | --- | --- |
|  | **BN ^z^** | **BN** | **BN** | **BN** | **Range** | **Skewness** | **Kurtosis** | **H^2^（%）** |
| 2014 | 106.8 ± 39.8 | 1.1 ± 0.1 | 1.3 ± 0.2 | 9.1 ± 34.4 | 1–249 | 4.8 | 25.1 | 62.8 |
| 2015 | 50.3 ± 3.6 | 1.0 ± 0.0 | 1.0 ± 0.0 | 1.8 ± 4.0 | 1–33.5 | 6.8 | 48.3 | 74.0 |

^z^ Means ± S.D.

**Supplementary Table 3.** QTLs for branch number detected by using the different reference sequences.

| **QTL** | **Position Interval (Mb)** | **10AS111A was used as the reference sequence** | | | **PI124039 was used as the reference sequence** | | |
| --- | --- | --- | --- | --- | --- | --- | --- |
|  |  | **SB’s SNP-index** | **HB’s SNP-index** | **Δ(SNP-index) ^a^** | **SB’s SNP-index** | **HB’s SNP-index** | **Δ(SNP-index) ^b^** |
| BN2.1 | 32.95–37.10 | 0.62 | 0.32 | 0.30 | 0.55 | 0.71 | 0.16 |
| BN2.2 | 39.80–42.25 | 0.73 | 0.33 | 0.40 | 0.53 | 0.70 | 0.17 |
| BN3.1 | 58.75–61.40 | 1 | 0.25 | 0.75* | 0.51 | 0.81 | 0.30 |

* Significant QTL at *p* < 0.05; ^a^ the peak Δ (SNP-index), calculated as (SNP-index of SB)–(SNP-index of HB); ^b^ the peak Δ (SNP-index), calculated as (SNP-index of HB)–(SNP-index of SB).

**Supplementary Table 4.** Sequences of primers used in the experiment.

| **Genes** | **Primer Name** | **Sequence 5’-3’** | **Usage** |
| --- | --- | --- | --- |
| *FALSIFLORA* (AF197936) | FA1F | AATTTCCTTTAAACGTTGTGTTTGT | Amplification of the gene |
|  | FA1R | AGGACATTCGTTCCACCGTC |  |
|  | FA2F | CGGCGAGTTTGTTCAAGTGG |  |
|  | FA2R | CACCTCCACCTCCTTGGTTC |  |
|  | FA3F | TCACTTTCATACCCCCACCAA |  |
|  | FA3R | ATGGTTGAGCCCATCAGTCC |  |
|  | FA4F | GGAGGTACATGGGAAGTGGC |  |
|  | FA4R | TCTTCGCGTACCTGAACACC |  |
| *COMPOUND INFLORESCENCE* (NP001234072) | SL1F | AGCTTTCTTATCTAGCTAGTCGCA | Amplification of the gene |
|  | SL1R | ACTACTTGCTTCGCACGTCA |  |
|  | SL2F | ATGCAACTGAAGTCATCCATTTT |  |
|  | SL2R | GGGTCCCACAAGATCGACAG |  |
|  | SL3F | CCATCCATGCGAACGTTTGTT |  |
|  | SL3R | TTCCTGTCTAACGAGCAGCC |  |
|  | SL4F | ACTCATGAGCTGTGACTGCAA |  |
|  | SL4R | AAGTCCAGAGCCAAAGCCTC |  |
| *FALSIFLORA* | FAP1F | ACTTTTAGTCAAAGGAATTGTGGTG | Amplification of the promoter region |
|  | FAP1R | GGCGAAGGGAGTAAAACACG |  |
|  | FAP2F | AGTCCTAAGGTTGACCACATCT |  |
|  | FAP2R | GGCGAAGGGAGTAAAACACG |  |
|  | FAP3F | TTGCACCACCATTTTTGAACTAAAG |  |
|  | FAP3R | GACACAACAGAGCTAAGGGGTT |  |
|  | FAP4F | GTGTTTTACTCCCTTCGCCTC |  |
|  | FAP4R | AACATTTTGCTCAAGTGGGGG |  |
|  | FAP5F | TGGAGCAATGAGTTCATGCAG |  |
|  | FAP5R | ATAGCAGGTAACCGTGTCCG |  |
| *COMPOUND INFLORESCENCE* | SLP1F | CTTTCCACGTCATCCCCTCC | Amplification of the promoter region |
|  | SLP1R | CACCAGTCGCAATGTCATGT |  |
|  | SLP2F | ACATGACATTGCGACTGGTGT |  |
|  | SLP2R | TGGTGTGCATCGACCTAAGTT |  |
|  | SLP3F | ATGACAACACGCTGTCTGCT |  |
|  | SLP3R | ATTCTGTGGGTACCCCTTCT |  |
|  | SLP4F | ACCCCCTGCATGACAAAAATA |  |
|  | SLP4R | CATGCGACTAGCTAGATAAGAAAGC |  |
| *FALSIFLORA* | snpfa1F | CCCCCACTTGAGCAAAATGTTC | HRM genotyping |
|  | snpfa1R | AACGTGCAAAGTTTACTAACAATCA |  |
| *COMPOUND INFLORESCENCE* | snps1F | ACGGAGACATAATTGATTTGGTACG | HRM genotyping |
|  | snps1R | TCAACATCAACAAATCTGCAGTC |  |
